# Sustainable cement-based composites: the effect of blended cements and CO_2_-cured ladle slag on electrical conductivity of silicate composites

**DOI:** 10.1038/s41598-025-30076-4

**Published:** 2025-12-03

**Authors:** Šimon Baránek, Vít Černý, Lenka Mészárosová, Jindřich Melichar, Rostislav Drochytka

**Affiliations:** Faculty of Civil Engineering, Institute of Technology of Building Materials and Components, Brno University of Technology, Veveří 95, 602 00 Brno, Czech Republic

**Keywords:** Silicate composite, Ladle slag, Graphite powder, Impedance, Leach impedance, CO_2_-curing, Energy science and technology, Engineering, Environmental sciences, Materials science

## Abstract

The cement industry is one of the largest environmental polluters, responsible for approximately 7–8% of global CO_2_ emissions. The search for more sustainable alternative binders and innovative composites is therefore becoming a key direction in the development of building materials. This paper investigates the effect of cement type and CO_2_ cured alternative binder on the resistivity of a silicate composite with carbon-based filler. The amount of electrically conductive filler was kept below the percolation threshold to observe changes in the electrical properties of the material due to chemical and microstructural modifications, and for the same reason the porosity of the material was maintained. The alternative binder was a 50/50 mixture of cement and grounded ladle slag, cured in a controlled CO_2_ climatic chamber. The results show that the use of CO_2_ cured binder not only helps to reduce the environmental footprint of the material but also contributes to the electrical conductivity parameters in the saturated state, which may be important for outdoor applications. The results further indicate that composites based on conventional CEM I and CEM II cements provide relatively good electrical conductivity, while furnace slag-rich CEM III cements show significantly reduced ionic conductivity and higher impedance, making them less suitable for electro-conductive applications. In contrast, the CO_2_-cured cement/ladle slag system demonstrated the highest leachate conductivity and the lowest impedance in the saturated state, confirming its potential as the most efficient and environmentally beneficial binder for outdoor electro-conductive composites. The study thus confirms that combining alternative binders with electrically conductive fillers can lead to more sustainable and functionally advanced building materials of the future.

## Introduction

Composite materials rank among the most advanced construction materials today and are widely applied across various industrial sectors. Their key advantage lies in the possibility to tailor their properties to a specific application. This is achieved through the selection and combination of different matrices and fillers. The matrix, forming the continuous phase of the composite, largely determines its physical and mechanical behaviour, as well as chemical resistance, thermal conductivity, fire resistance, and other characteristics^1^. Commonly, matrices are based on silicates, polymers, or geopolymers. Fillers, on the other hand, typically help lower production costs while also influencing bulk density, electrical conductivity, absorbency, and related properties. These advanced materials have applications in smart infrastructures, such as monitoring traffic loads and detecting failures in road structures or bridges, ohmic heating paves and bridges^2–4^.

Although the matrix may not be the main element of the electrical conductivity, its proper electrical conductivity can contribute significantly to reducing the cost of composite production^5^.

Since the cementitious composites always acts like a poor semiconductor or insulator due to its high resistivity, which ranges from 10^3^ Ω·cm (for water-saturated concrete) to 10^9^ Ω·cm (resistivity of cement itself is usually 9—10·10^8^ Ω·cm)^6^, for effective heating, resistivity values in the range of 400 to 1500 Ω·cm are recommended, which are significantly lower than the values of the cement matrix^7,8^. Variables such as water to cement ratio, cement to sand ratio, moisture content and electrode size and spacing were also studied^9^.

Most of the research mainly focuses on the solution of electrically conductive fillers, in particular (amount, type of filler or behaviour in external conditions such as humidity, temperature, dynamic cycles in frost, de-icing, etc.), and mainly uses common Portland cement (PC) as the silicate matrix or pure primary matrix. For example, Tao Y.et al. discusses for PC based sensors the use of carbon fibre reinforced polymer recyclate on the electrical resistivity and external impact resistance of piezoresistive cement composites under varying temperature, humidity and chloride-induced corrosion using cement with Portland clinker content above 97%^10^. For example, Zhang B. et al. studied bitumen-based matrices using graphene paper for snow melting of pavements I at—30 °C^11^.

The problem with the lack of electrical conductivity of the silicate matrix (cement putty) is mentioned by Rui Rao et al. They mention that hardened cement between carbon or steel fibres limits the tunnelling effect of electrons and prevents the steel fibres from forming a continuous electrically conductive network. They mention the use of finer fibres by increasing the aspect ratio as one way to mitigate this effect but conclude that by selecting the appropriate type of matrix/cement, this negative effect could be effectively minimized as a possible future research direction^12^

Furthermore, Rovnaník P. et al. mention in their research that matrix from alkaline activated slag shows better electrically conductive properties due to the presence of mobile hydrated sodium ions and metallic iron microparticles. They also compared the changes in resistivity of cement matrix (0.5 Ω·cm) and alkaline activated matrix (397 Ω·cm) under 45 kN loading for sensing purposes, therefore, AAC has better potential for use in sensor technology^13^.

However, most research focusing on electrically conductive composites (ECC), works with pure PC—CEM I or with a low amount of fly ash admixtures^14,15^. Very few authors use slags, for example Pereira N. et al. studied mortars and the use of foundry slags as sand replacement in their research and found that replacing foundry slag reduces impedance by approximately 20%, which also points to the potential use of CEM II/A-S and CEM II/B-S cement for electrically conductive composites, but foundry slags contain more free ions than blast furnace slags or steel blast furnace slag (SBFS). Although the resistivity of cement itself is usually around 9—10·10^8^ Ω·cm, it is possible to achieve much lower values thanks to blended cements^16^.

However, research is also focusing on ecology in ECC designs. In their 2025 research paper, Lu J. et al. state that future research in this area should focus primarily on the use of secondary electrically conductive raw materials (carbon fibres) and on improving the electrical conductivity of all phases of conductive concrete, i.e., including binders, with regard to the environment^17^.

Another key parameter affecting electrical conductivity is the density of the material. To achieve a dense silicate composite, it is necessary to use plasticizing or super-plasticizing additives. On the one hand, if the matrix itself is highly conductive, it offers the composite better electrical conductivity than the pores it contains. The porosity of the composite can also be reduced by adding fine-grained admixtures. By mixing both fly ash and slag, the resistivity was even increased more. Some studies also indicate that mixing fly ash or slag with PC can further increase resistance. Such an increase in resistance has been attributed to the densified microstructure of the C–S–H gel and modified ion species and concentrations. Slag hydrates and causes a pozzolanic effect. The hydration products of C–S–H and C–A–H gels fill micropores, but are non-conductive, thereby reducing porosity and pore connectivity^9,18^.

Geopolymers containing slag, fly ash and polymers were all studied as matrices for ECC. They also state that alkali-activated industrial by-products, such as blast furnace slag, have comparable or even better properties than ordinary Portland cement also in electrical conductivity parameters^19^. Compared to conventional concrete, geopolymer concrete acting like a superionic conductor via Na^+^ ion hopping. The conductive mechanism changes from electron hopping to the combination of electron hopping and conductive pathway after the presence of conductive filler thus results in the reduction of composite resistivity^1,2,20,21^.

Among all these materials, Portland cement is the most widely used material. Thus, cementitious composite involved cement paste, mortar or concrete is the most common matrix material, highly depends on the dispersion of electrically conductive fillers, the water to cement ratio, sand and aggregate content, and the addition of chemical admixture may affect the composite rheology and govern dispersion of conductive fillers. Besides that, the supplementary cementitious materials such as silica fume and fly ash may also affect the electrical properties of the cementitious composite^7,21–23^.

Studies by Liu K et al. reported that when cement is mixed with water, ions such as Na^+^, K^+^, Ca^2+^, SO_4_^2-^ and OH^-^ are dissolved into the pore water, which increases the concentration of ions in the pore water. These ions potentially increase the electrical conductivity of the electrolyte but are partially adsorbed to the C-S–H structures and also presents a good electrical conductivity due to the low resistivity of FeO and Fe_3_O_4_, which is around 5·10^–2^ and 4·10^–3^ Ω·cm respectively^24^.

They also mention, and several studies agree, that the electrically conductive properties of composites depend mainly on the nature, amount, distribution and appropriate shape of the electrically conductive filler particles. The suitability of these particles for forming a complete electrically conductive network can be evaluated experimentally by optical microscopy with polarizing filters on sections or on the fracture surface of the test specimens. Additional suitable methods are the scanning electron microscope methods Energy Dispersive X-ray (EDX) and Wavelength-dispersive X-ray spectroscopy (WDX), which are able to detect up to 95% of the elements of the periodic table, including trace amounts, even over an area of a few µm^2^^24,25^.

Therefore, there is also pressure throughout the construction industry to use secondary materials such as slags (blast furnace slags, ladle slags), waste biomass, fly ash (fluidised, high temperature, bedding), waste limestone, etc.^26,27^. The slag from the ladle (LS) is a by-product of the secondary processing of steel. Typical processing of LS is storage in open landfills. As the production of LS is relatively high, a lot of studies and research has already been focused on how to utilise this raw material. The construction industry is a potential area that can recycle LS as a sustainable substitute for cement binder, thereby reducing cement consumption, conserving natural resources and mitigating greenhouse gas emissions^28,29^. Najm et al. present in their study the possibilities of using LS in the concrete industry. Modifying the properties, grinding, sieving and adding gypsum from pan slag significantly improves its bonding properties. It also lists applications for alkali-activated materials where it depends on the curing temperature and the type and components of the alkali activator solution. Also, the replacement of 20% of cement with pan slag does not compromise the strength and durability aspects of cement-based concrete^28^. Sáez-de-Guinoa et al. have studied the addition of pan slag (up to 39% addition of raw slag to clinker) already in the production of Portland clinker and found that it positively affects the mechanical properties^30^. A study by Shi, states that slow cooling of LS leads to a crystalline microstructure with conversion of β-C_2_S to γ-C_2_S^31^. The ladle slag has very weak pozzolanic properties, however, the potential property of fine LF slags increases significantly with fineness regardless of some differences in their mineral composition or when an activator is added^31–33^.

Another point in this article is the reduction of CO_2_ pollution, as global sustainable development faces challenges in terms of consumption of non-renewable resources and energy, landfill waste and pollution, as well as greenhouse gas emissions^34^. Two of the biggest polluting elements in the construction industry are cement production and steel production. Cement production accounts for approximately 7–8% of global CO_2_ emissions, equivalent to approximately up to 2.6 gigatonnes per year, with 0.6 tonnes of CO_2_ emitted per 1 tonne of cement produced^35–37^. Producing one tonne of steel generates, on average, about 1.85 to 1.9 tonnes of CO_2_. The global steel industry is a significant contributor to greenhouse gas emissions, responsible for roughly 7–9% of total human-made CO_2_ emissions worldwide^38,39^. In this study, we focus on the use of ladle slag, which has potentially suitable properties for both CO_2_ treatment and electrically conductive parameters. The high calcium content in the LS makes it CO_2_-reactive. The slag and CO_2_ gas reaction will produce calcium carbonate (CaCO_3_) and weak carbonic acid. CaCO_3_ can be used as a binder in construction^32^. The absorption of CO_2_ significantly depends on the quantity of slag and its CaO content^29,31^. Mineral carbonation has certain advantages, such as the thermodynamic stability of the resultant product and lower costs due to lower energy consumption because of the carbonation reaction’s exothermic nature, along with the storage’s permanency and safety. The chemistry of LS depends on the batch of steel and is inconsistent^40^. However, LS typically has a high CaO content of 50–60%^32,41^.The LS showed a CO_2_ sequestration potential of approx. up to 25 g of CO_2_/100 g of LF slag (under ideal conditions—unrealistic), and portlandite gives a high CO_2_ sequestration potential where theoretical values are up to 60g/ 100g of pure portlandite^42–44^.

The use of the carbonation process for curing CaO-based binders is already quite well known but not very much used in practice. Carbonation of concrete is a chemical process caused by the reaction of CO_2_ with the cementitious cement components in the concrete, which gradually lowers the pH of the pore solution^45^. The CO_2_ molecules penetrate uniformly from the surface of the concrete to its inner layers by diffusion. The (OH^-^) in the pore solution is formed both by the dissociation of Ca(OH)_2_, which is formed by the hydration of silicate clinker minerals, and by the dissociation of NaOH and KOH, which are formed by the hydrolysis of Na_2_O and K_2_O-containing minerals. While sodium and potassium hydroxides are completely dissolved in the pore solution which potentially increases the electrical conductivity of ECC. In summary, the carbonation process involves the diffusion of carbon dioxide in the gaseous phase through the pore structure of the material, curing CaO + CO_2_ → CaCO_3_^46,47^. The CO_2_ curing can thus significantly improve the environmental aspects of building materials production and also contribute to improving the properties of existing conventional products.

This paper thus addresses the existing gap in knowledge regarding the influence of blended cement type on the performance of electroconductive cementitious composites (ECC), including a comparison with CO_2_-cured alternative binders. The study builds upon previous research highlighting the potential of blended cements and carbonation curing to enhance functional and sustainability aspects of cement-based materials^48–50^.

## Materials

The experiments were verified on simple mixtures (only cement, carbon powder and water), due to elimination of possible negative variables that may affect the electrical conductivity of the composite. The composition of these prototype composites is also based on previous research that has shown that the addition of non-conductive aggregates and additives will only increase the resistivity of the composites to the same extent for all types of mixtures according to previous research^20^. The experiment was performed on 9 specimens from each mixture (108 in total), with the most extreme results excluded from the analysis.

### Binders

**Fig. 1:**
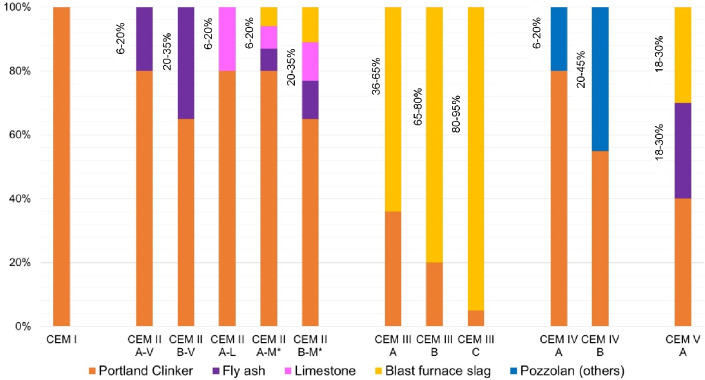
Summary of cement compositions according to EN 197. *CEM II A-M / B-M - The possibility of one type of admixture or combinations thereof.

Locally available mixed cements were used as binders and pure Portland cement CEM I was used as reference. An overview of the composition of blended cements by designation are shown graphically in Fig. 1.

Ladle slag (LS) was used as an alternative CO_2_ -cured binder. In order to achieve suitable handling strengths so that the material could be transported to the CO_2_ climate chamber, the mixture was adjusted to a mixture of 50% Portland cement CEM I and 50% LS.

LS unlike blast furnace slag, which comes from core iron production, it is a product with a variable chemical and mineral composition, depending on the type of steel processed and the additives used. It typically contains a high proportion of CaO and Al_2_O_3_, as well as SiO_2_, MgO, smaller amounts of FeO, MnO and other oxides as you can see in Table 1. Adverse effects on building materials are mainly due to volumetric changes caused mainly by the free CaO content and the characteristic presence of calcium silicate (γ-C_2_S), which can hydrate and cause volumetric instability (dust effect). Therefore, LS is generally considered to be less stable than blast-furnace granulated slag and its use in the cement or construction industry is limited. It is therefore often used after treatment—e.g. ageing, CO_2_—curing or heat stabilisation. The used LS was micro-grounded to a specific surface of 3500 cm^2^/g.

**Table 1:** Chemical composition of the binders.

Oxide:	CEM I 42.5R	CEM II B-M (V-LL) 32.5R	CEM II BS 32.5R	CEM II AS 42.5 R	CEM II A-LL 42.5 R	CEM II A-S 52.5N	CEM II A-LL 52.5R	CEM III B-(LH/RS) 32.5L	CEM III A 42.5N	CEM I 42.5R + LS
CaO	64.236	47.845	48.694	52.311	54.663	51.140	53.995	41.020	47.118	48.316
SiO_2_	19.487	18.234	24.184	19.127	14.842	18.529	15.611	28.791	25.075	15.416
**Fe**_**2**_**O**_**3**_	**3.345**	**3.379**	**2.639**	**2.859**	**2.854**	**2.302**	**3.148**	**1.539**	**1.781**	**7.966**
Al_2_O_3_	4.791	5.952	4.641	4.186	3.661	4.016	3.545	6.054	4.949	5.939
MgO	1.310	1.099	3.147	2.198	1.131	3.338	1.137	6.706	3.162	5.019
MnO	0.289	0.228	0.467	0.311	0.098	0.270	0.248	0.628	0.238	1.127
**Na**_**2**_**O**	**0.126**	**0.147**	**0.414**	**0.250**	**0.251**	**0.351**	**-**	**0.422**	**0.419**	**0.189**
**K**_**2**_**O**	**0.438**	**0.516**	**0.471**	**0.443**	**0.403**	**0.307**	**0.453**	**0.462**	**0.430**	**0.356**
SO_3_	2.247	1.886	2.280	2.196	1.891	2.347	2.115	2.166	2.147	1.488
I	1.113	1.223	1.134	1.436	1.146	1.302	1.332	1.190	1.210	1.273

The following Fig. 1. summarizes the composition and classification of blended cements according to EN 197–1^51^.The classification is divided chronologically according to the type and quantity of admixtures, or combinations thereof, with the subsequent number indicating the strength class (32.5, 42.5, and 52.5) and the last letter indicating the speed of strength increase (L – low , N—normal, R—rapid). There is also specific labelling, for example LH, for cements with low heat of hydration or SR for sulphate-resistant cements.

Table 1 shows the chemical analyses of used binders, the main components that affect the electrical conductivity are marked in bold. According to the chemical analysis of the binders, it can be seen that mixtures containing ladle slag contain up to 3 times more iron oxides than cement mixtures, while it contains a smaller amount of alkali that can potentially release alkali K^+^ and Na^2+^ ions.

The chemical composition of used binders is given in the following Table 1.

Generally, blended cements are finer ground to achieve the required strength characteristics and to activate the admixtures. The mixture with ladle slag has the lowest specific surface, as the slag is ground to approximately 3600 cm^2^/g.

The specific surface area of the binders used is given in Table 2.

**Table 2:** The specific surface of the binders used.

Binder:	CEM I 42.5R	CEM II B-M (V-LL) 32.5R	CEM II BS 32.5R	CEM II AS 42.5 R	CEM II A-LL 42.5 R	CEM II A-S 52.5N	CEM II A-LL 52.5R	CEM III B-(LH/RS) 32.5L	CEM III A 42.5N	CEM I 42.5R + LS
Specific surface area [cm^2^/g]	4010	5400	4430	5230	5270	5500	5650	4430	4970	3850

### Electro conductive filler

**Fig. 2:**
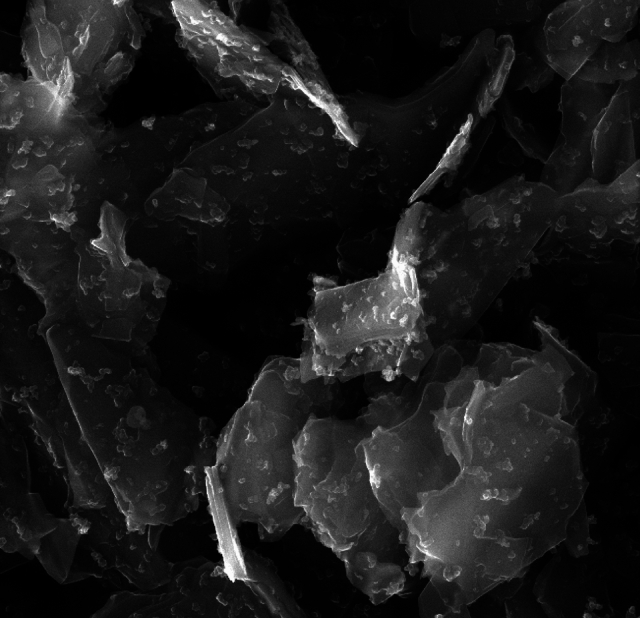
Detailed SEM image of used Condufit C4 graphite particle surface with nanoflakes – magnification 20,000 × . (author’s photo).

Condufit C4 graphite powder was used as the electrically conductive element. Condufit C4 is micro-milled natural graphite with a specially modified surface using nanoparticles “nanoflakes” that increase electrical conductivity see Fig. 2. It consists of 99.5% carbon and a maximum of 0.5% ash. Maximum moisture content is 0.5%. Particle size D(0.5) = 2.9 μm, D(0.9) = 5.2 μm.

### Super plasticizer—ViscoCrete®-1035

An effective superplasticizer based on polycarboxylates was used to achieve good workability and compatibility of the mixtures. ViscoCrete 1035 from Sika company is a water-reducing concrete admixture according to EN 934–2^52^. A dosage of 0.5% of the binder weight was used.

### Composition of mixtures

The mixtures on which the electrical conductivity parameters of cements have been studied consist of cement, a small amount of carbon, water and a superplasticizer; mixes with an alternative CO_2_-cured binder contain cement substitution using ladle slag. For mixtures in which CO_2_ curing was used, the different water ratio (0.40/ 0.45/ 0.50) was adjusted to achieve a porosity and internal structure similar to that of samples made with cement binders. The aim was to achieve similar porosity of the materials by using mixing water, so that the resistivity values would be comparable.

The detailed composition of the mixtures is shown in the following Table 3.

**Table 3:** Composition of the mixtures for testing the effect of cement type and CO_2_ cured alternative binder on the electrically conductive properties.

Binder type :	Cement [%]	LS [%]	Graphite [%]	w/c ratio	Flow value [ø mm]
CEM I 42.5R	96	-	4	0.50	150
CEM II/B-M (V-LL) 32.5R	96	-	4	0.50	150
CEM II/B-S 32.5R	96	-	4	0.50	155
CEM II/A-S 42.5R	96	-	4	0.50	145
CEM II/A-LL 42.5R	96	-	4	0.50	155
CEM II/A-S 52.5N	96	-	4	0.50	145
CEM II/A-LL 52.5R	96	-	4	0.50	145
CEM III/B (LH/SR) 32.5L	96	-	4	0.50	150
CEM III/A 42.5N	96	-	4	0.50	150
CEM I 42.5R + LS *	48	48	4	0.40	145
CEM I 42.5R + LS *	48	48	4	0.45	155
CEM I 42.5R + LS *	48	48	4	0.50	165

* *Mixtures that were placed in a CO*_*2*_* climate chamber immediately after hardening (4 h).*

## Methods

Based on previous research, the following set of methods was chosen to study the influence of individual binders in terms of electrical conductivity. These are methods focused on the analysis of materials in terms of physical and mechanical parameters and in terms of electrically conductive properties—impedance. For composites, the main parameters measured were porosity, bulk and specific weight and impedance. Furthermore, chemical analysis by XRF was performed on the hydrated composites and the leachate impedance was measured.

### Preparation and production of test specimens

The materials for the composites (cement and graphite powder) were dry homogenized for 5 min in a 3D powder mixer (Turbula-type) to ensure uniform distribution of the conductive filler.

Then, 3/4 of the premixed water was added, followed by the super plasticizer and the rest of the premixed water to the required consistency.

The consistency of fresh mortar was then measured according to EN 1015–3, Part 3: Determination of the consistency of fresh mortar (according to the flow table test the required consistency flow value was 150 ± 10 mm)^53^.

**Fig. 3:**
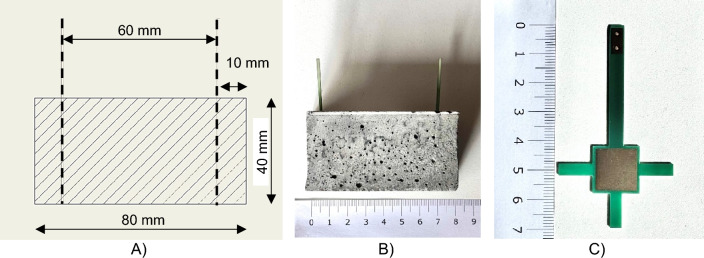
(**A**) Scheme of the testing specimen for impedance measurement, (**B**) photo of the specimen, (**C**) detail photo of used electrode.

The mixture was then filled into 40 × 40 × 160 mm, moulds according to EN 196–1 and compacted on a vibrating table^54^. For measurement of electrical conductivity parameters, electrodes with a printed junction and a contact area of 196 mm^2^ were used. For impedance determination were the specimens fitted with four electrodes in the fresh mixture, see Fig. 3 , at distances of 1,7,9 and 15 cm from the edge of the mould. This resulted in two test specimens 40 × 40 × 80 mm, with two electrodes at a distance of 60 mm.

### Storage of specimens and CO_2_—curing

The cement-based specimens were then placed in an aqueous environment where they hydrated for 28 days. After 4 h, the samples with C/LS content were transferred to a CO_2_ conditioning chamber- Thermo Scientific BBD 6220 CO_2_ Incubator for a 72h with setup:

- CO_2_ concentration = 20%,
- RH = 60%,
- temperature = 28.5°C.

The samples were also weighed in a fully saturated state before being placed in the CO_2_ climate chamber, so that the amount of CO_2_ captured by the composite could be approximately determined.

### Density

“True” density was determined using a helium pycnometer—AccuPyc II 1340. This pycnometric method uses the inert gas nitrogen to determine the specific gravity. This method is more accurate than methods using water or alcohol as the measuring medium. this method is based on standard EN 1097–7^55^.

### Bulk density

The bulk density of a material is defined as the ratio of the weight of the material to its total volume, including all pores and voids. The determination of the bulk density was carried out by gravimetric method according to EN 12390–7^56^**.**

### Porosity, water absorption

The porosity ($\varnothing$) of the hardened composite was determined indirectly from density measurements. First, the true density (ρ_m_) of the powder fraction was determined according to EN 1097–7^55^. Subsequently, the bulk density (ρ_bulk_) of the fabricated test bodies was determined according to EN 12390–7^56^. From these data, the total porosity was calculated according to equal:

$$\varnothing =1-\frac{{\rho }_{bulk}}{{\rho }_{m}}$$

The water absorbency (W) of the composite was determined by gravimetric method according to standard ČSN 72 1326^57^. The absorption calculation is based on the weight difference between the saturated sample (m_s_) and the dry sample (m_d_) relative to the dry weight:

$$W=\frac{{m}_{s}-{m}_{d}}{{m}_{d}}$$

### X-ray fluorescence (XRF)

XRF analysis was used to determine the chemical composition of the binders. XRF is a method based on X-rays or gamma rays, irradiating the sample, which excite electrons in the inner shell of the electron shell of the atoms in the surface layer of the material under investigation, creating a so-called photo effect. This X-ray fluorescence energy is measured, and its value is then compared with the values characteristic of the individual elements that could be found in the material^58^.

### Specific surface area

The specific surface area of the binder was determined by the air permeability method using a Blaine apparatus, in accordance with EN 196–6:2018 – Methods of testing cement – Part 6: Determination of fineness. Equivalent requirements are also provided in ISO 5790:2023 – Measurement of fineness of hydraulic cement by air permeability apparatus^59^.

### Impedance of hardened composites

**Fig. 4:**
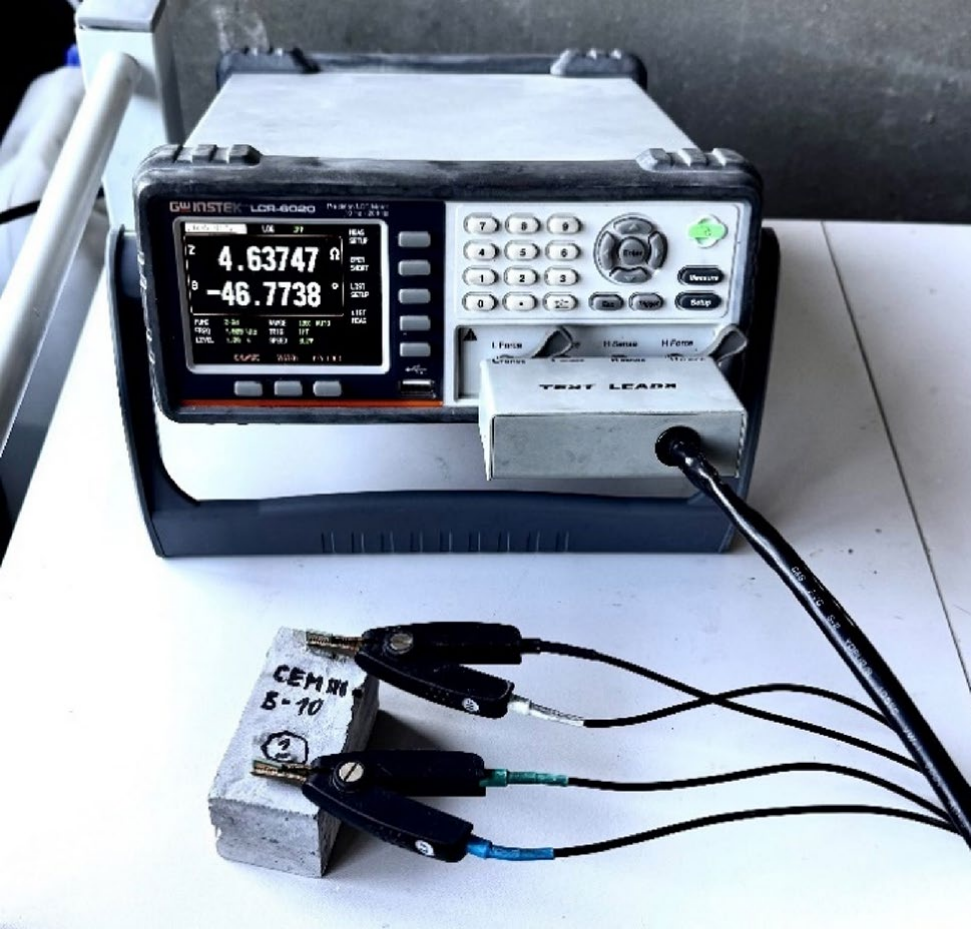
Measurement of the two-point impedance of a composite specimen using the GW Instek LCR 6020.

The simplest method was chosen to compare the electrical conductivity parameters of the composites—impedance measurement, as the test specimens had the same dimensions, including the distance and area of the electrodes. To measure the electrically conductive parameters—Impedance, the GW Instek LCR 6020 was used which measures the impedance [Ω] and the phase angle in [°] which describes the phase movement between the real voltage and current. The impedance determination of the test samples was performed using a methodology related to previous research^20^. The impedance is measured by direct connection of the sample electrodes to the contact cables of the instrument, see Fig. 4.

### Impedance of the leach

In order to specify and compare the electrical conductivity parameters in the saturated state of the materials, the electrical conductivity of the leachates (electrical conductivity of the electrolyte of the specimen) was determined. This method was derived from the determination of the electrical conductivity of the leachate according to EN 13038 but is only used for comparison in research.

**Fig. 5:**
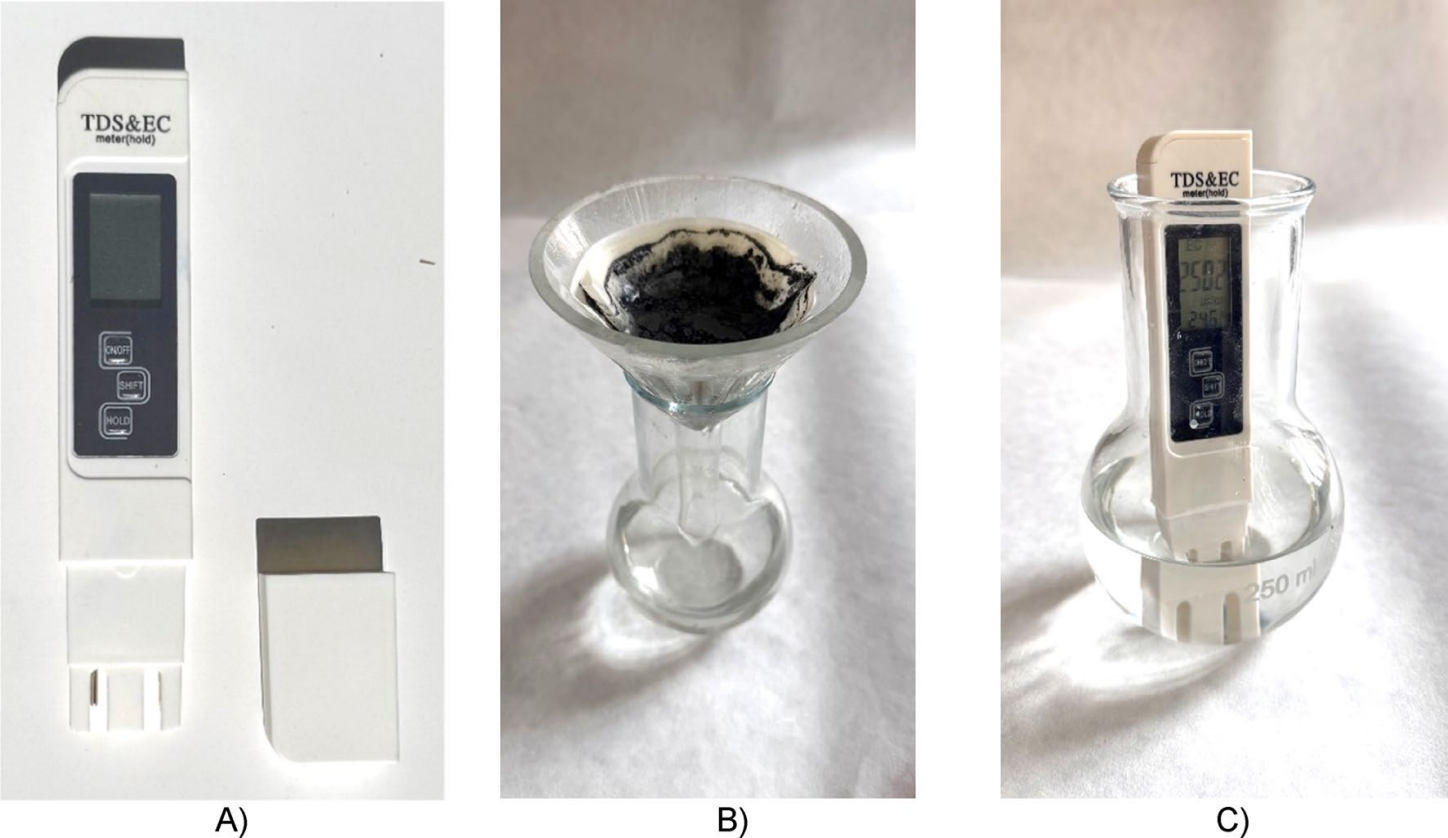
(**A**) E-1 portable TDS & EC Meter, (**B**) filtration of leachate after 24 h, (**C**) measurement of the electrical conductivity of the leachate.

The leach was carried out in a 1:10 ratio (25 g of finely ground dried hydrated sample and 250 ml of distilled water) in a laboratory condition 23 ± 2°C. The leaching time was 24h, then the leachate was filtered through filter paper see Fig. 5, and the electrical conductivity was determined using an E-1 portable TDS & EC Meter in [μs/cm] with accuracy: ± 2%.

## Results and discussion

The main objective of this paper is to compare different types of cements and their CO_2_ friendly alternatives for electrically conductive composites. The main parameters for comparison were the impedance of the material itself and the impedance of the leachate from the material, which simulates the resulting electrolyte when the material is saturated with water.

To make the results comparable, the same manufacturing procedures were used and as far as possible the same internal structure of the composites was achieved, in particular the porosity and density of the material.

### Comparison of the internal structure of composites

**Fig. 6:**
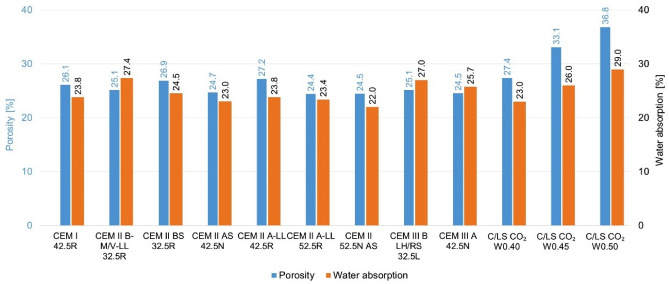
Graphical comparison of porosity and water absorption of composites.

**Fig. 7:**
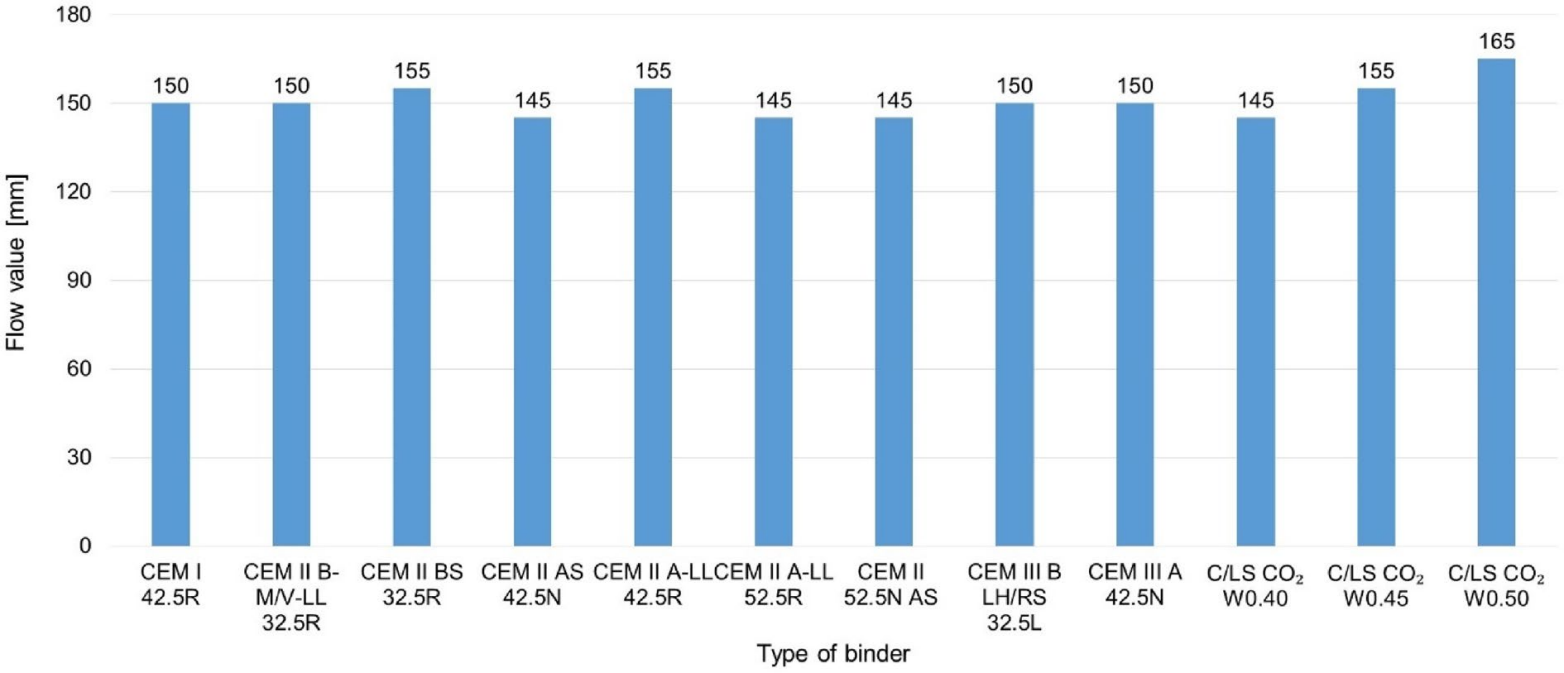
Chart comparing the consistency of the mixture (Flow value).

**Fig. 8:**
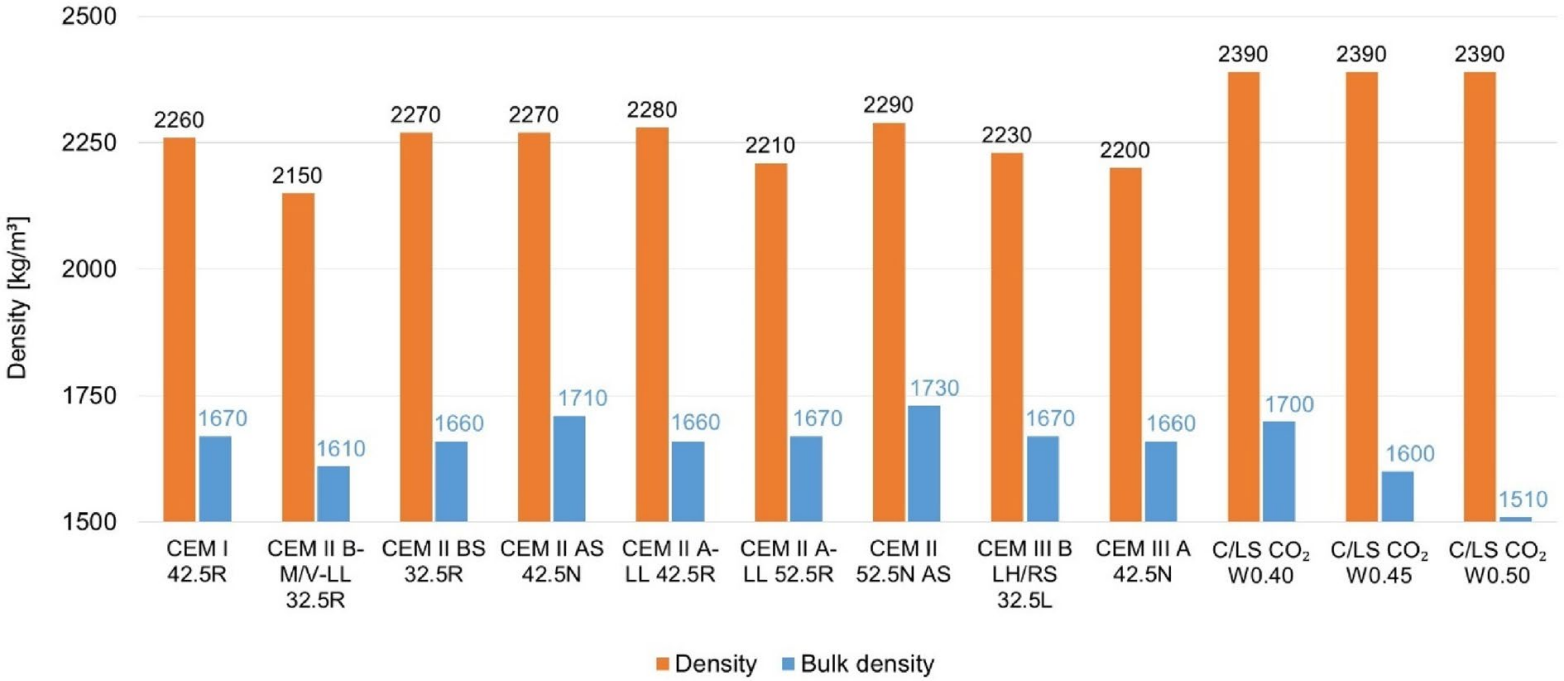
Graphical comparison of true and bulk density of composites.

Maintaining the same internal structure, in particular the porosity of the materials, is an important parameter for comparing the electrical properties of the composite or if they significantly affect the electrical conductivity. To achieve the same internal structure, it was optimized to the same consistency of the mixture by flow table. For mixtures with C/LS (CO_2_ cured binder), a step water coefficient was chosen, and for a w_c_ of 0.4, a similar internal structure to that of pure cement composites was achieved see Fig. 6. For the true density and bulk density values, comparable structures were also obtained, only for the CO_2_ cured mixtures the true density is slightly higher as the material is denser after carbonation. From the results, the alternative mix C/LS CO_2_ W_c_0.40 was again the most suitable for comparison, see Fig. 7 and 8.

Based on the evaluated parameters of bulk density and specific weight, water absorption, total porosity, and consistency of the fresh mixture, it is clear that the individual recipes have different internal structures. The reference cement mixtures achieve relatively balanced values of porosity (approx. 24–27%) and water absorption (23–27%), which is also reflected in a stable bulk density of around 2200–2280 kg/m^3^. In contrast, C/LS mixtures with a water coefficient of 0.40–0.50 show a higher variability – while at w/c = 0.40 the porosity and water absorption values are still comparable to the reference, at w/c = 0.45 and especially 0.50, there is a significant increase in these parameters (porosity up to 36.8%, water absorption 29–37%). This trend is also confirmed by a decrease in bulk density (down to 1510 kg/m^3^), which indicates a thinner and more porous structure. In terms of the consistency of the fresh mixture (Flow table test), it is apparent that a higher water coefficient leads to higher flowability, which correlates with an increase in porosity and reduced compactness of the hardened matrix.

**Fig. 9:**
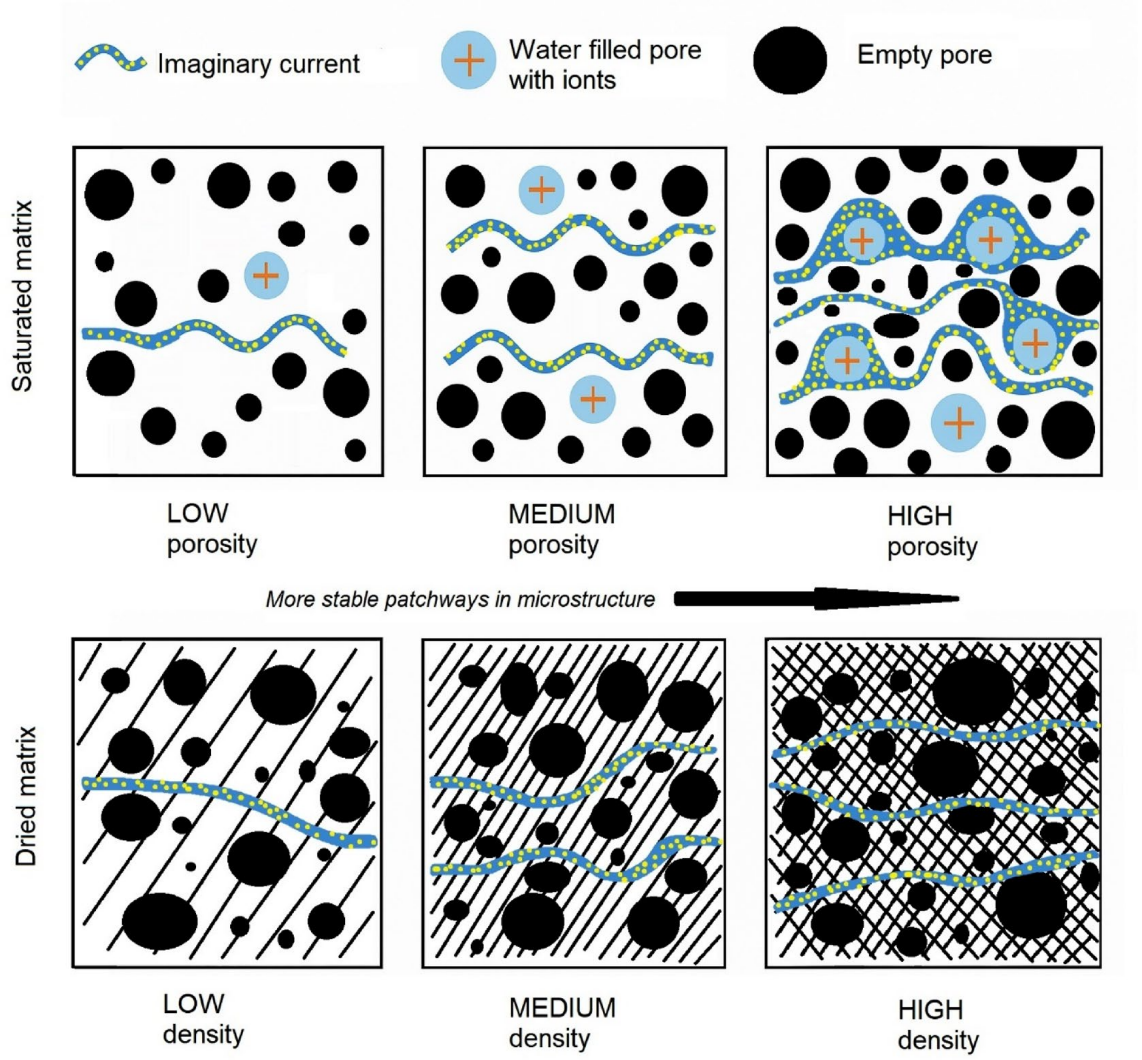
Schematic representation of the influence of internal structure on the electrical conductivity of composites. (author’s image).

The influence of microstructure and saturation on electrical conductivity parameters is shown in Fig. 9.

In Fig. 9 the upper set of images shows the influence of porosity during water saturation – a higher pore volume also means a higher number of pores that can be saturated with a pore solution (depending on the permeability of the matrix—this explains the difference in porosity and water absorbency measured above in Fig. 6.), which allows ion current to flow through the pore water phase. The bottom set shows the effect of matrix density in the dry state, where higher density of the material itself and also the proportion of electrically conductive filler ensures better electrical conductivity.

### Impedance of composites

**Fig. 10:**
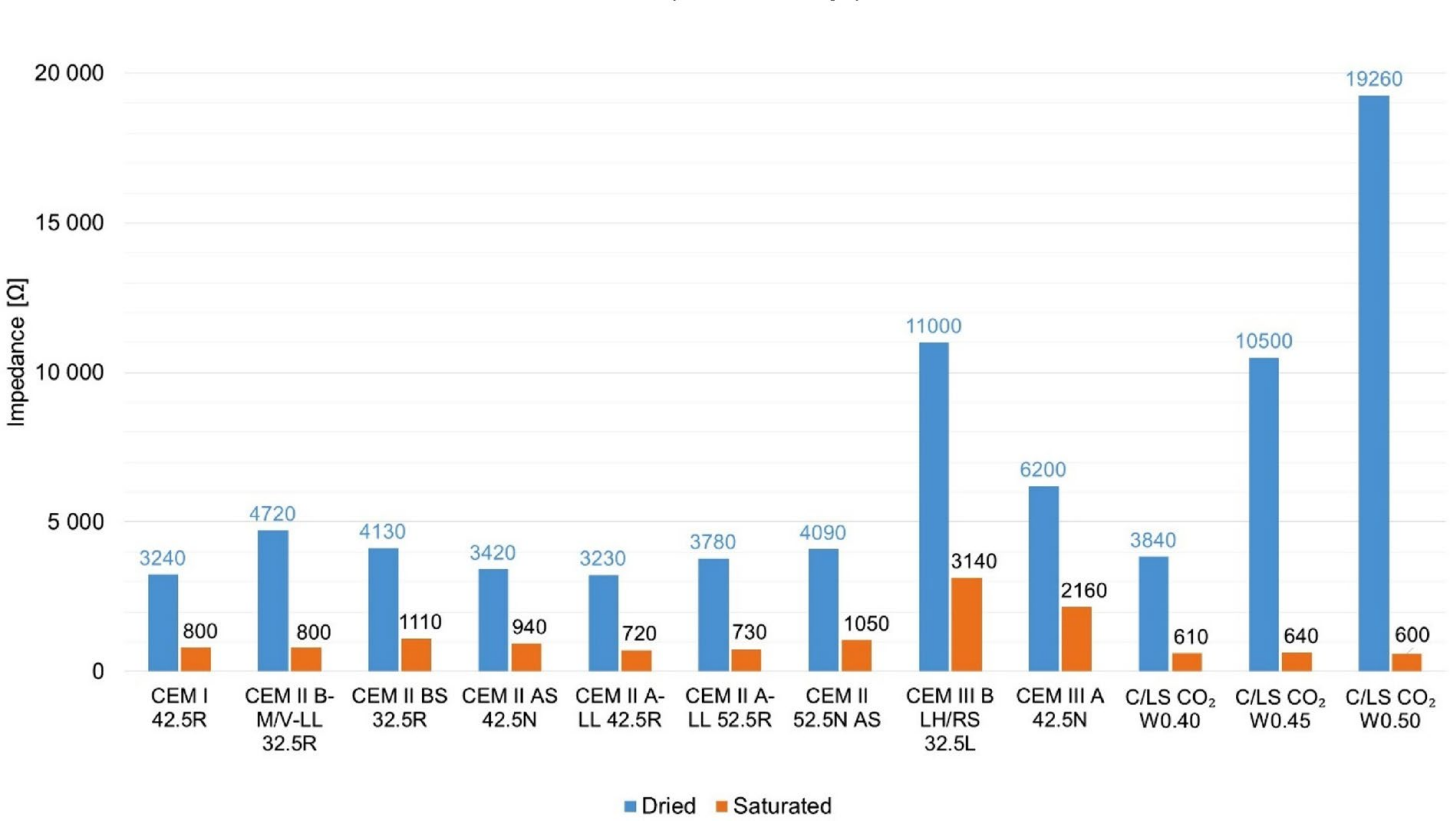
Graphical comparison of impedance of composites.

The lowest impedance is achieved by mixes with CEM II A-LL 52.5R (2780 Ω) cement with 6–20% limestone and CEM I 42.5R (3240 Ω) cement—these mixes achieved the best electrical conductivity of the standard mixes. In contrast, the CEM III B LH/RS 32.5L (11,000 Ω) cement with 66–80% granulated blast furnace slag has significantly higher impedances. The comparison of impedance of composites with different type of binder is shown graphically in Fig. 10 below.

Samples in a dry state the alternative cement replacement (C/LS W0.40 + CO_2_ curing) achieved slightly better values (3840 Ω) than the average standard cement.

An interesting result is the mixture C/LS W0.40 + CO_2_ curing, as it reached the best electrical conductivity in the saturated state, this phenomenon is probably due to free ions that are doped into the electrolyte by the slag itself.

The above results show that only the C/LS mixture with a water coefficient of w/c = 0.40 was selected for the next experimental part. This variant shows the closest values of porosity, water absorption, and bulk density to the reference mixtures and at the same time provides a more stable internal structure, which allows for a relevant comparison of results. At the same time, this formulation can be expected to have more favourable transport (electrical conductivity) properties due to its limited porosity and better connectivity of the pore network. In the following text, this mixture is therefore **referred to only as C/LS**.

Based on available publications and experience with matrix hydration reactions, it can be concluded that upon contact with water, the slag excretes Ca^2+^, Mg^2+^ (less) and accepts Fe^2+^/Fe^3+^ ions, possibly small amounts of hydroxyl Al^3+^ or Si ions, releasing hydroxides because the system is strongly basic^60^. However, these are mostly bound to CaCO_3_ during hydration, (Ca^2+^ and CO_3_^2-^) in the crystal lattice. CaCO_3_ is very slightly soluble in water; the solubility product is K_sp_ ~ 4.55 × 10^–9^ at 25 °C, corresponding to a maximum solubility of about 6.8 mg/l for equimolar Ca^2+^ and CO_3_^2-^ ions. Such leaching of Ca^2+^, Mg^2+^, Fe^2+/3+^ are probably not from carbonates and increases the conductivity of the electrolyte, which may explain the significantly lower impedance in saturated state measurements for slag mixtures.

We can also see (Table 1) that the alkali content in slag is lower than in conventional cements, probably K^+^, Na^+^ are either not released or not present in such quantities to affect the electrical conductivity of the composite or do not enter the electrolyte in free form. Furthermore, CO_2_ -curing leads to carbonation of portlandite (Ca(OH)_2_ → CaCO_3_) or Ca/Mg hydroxides. Carbonates (e.g. CaCO_3_) do not conduct ions as well—the result is the formation of a dense layer that gradually closes the pores and reduces the electrical conductivity of the system. The carbonate phase CaCO_3_ is non-conductive and contributes to the confinement of the microstructure, which reduces ionic mobility and increases dry impedance. The low solubility means that CaCO_3_ does not form a conducting solution and therefore has no significant ionic contribution^61–63^.

### Impedance of the leach

**Fig. 11:**
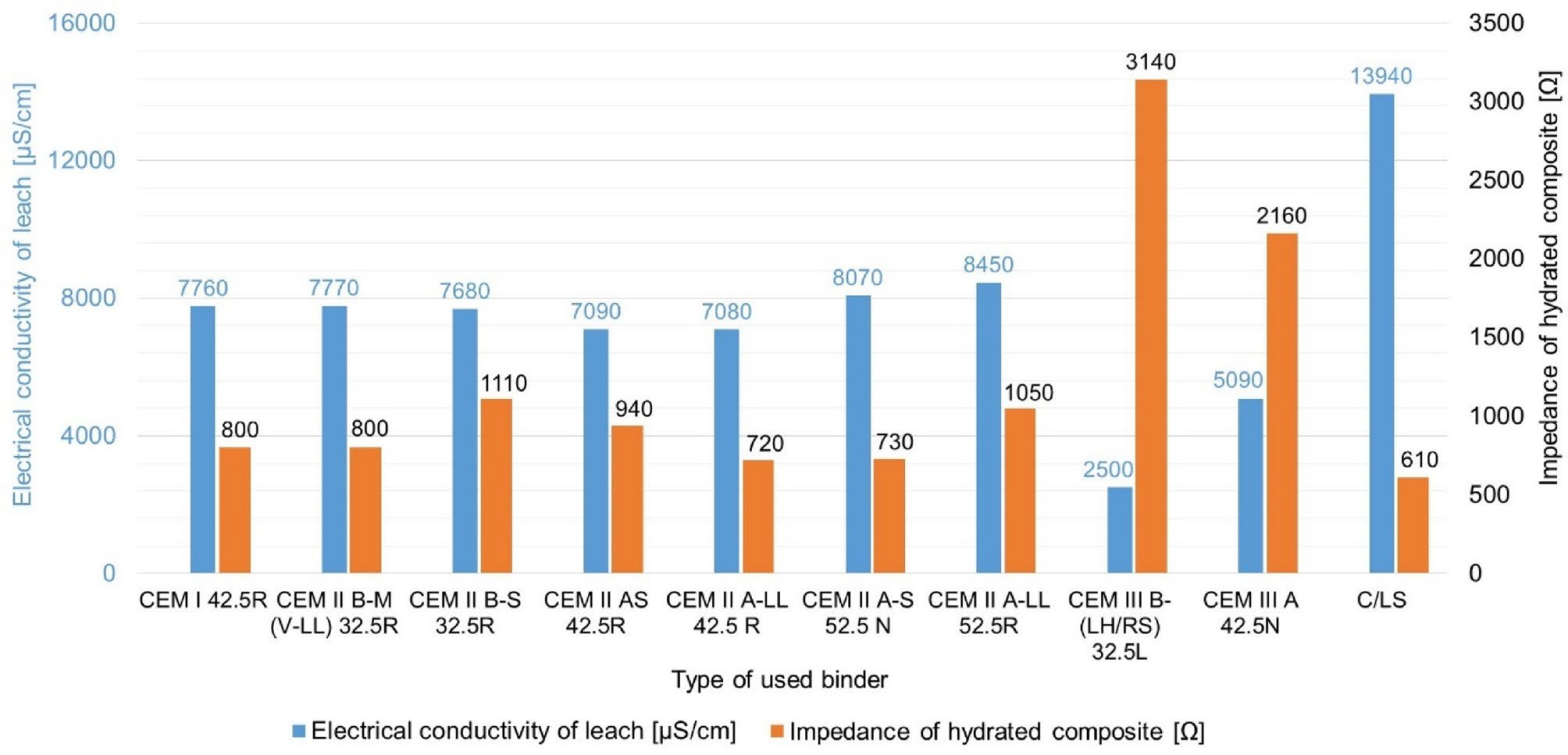
Comparison of the impedance of hydrated composites in the saturated state and the electrical conductivity of the leachate.

The measurement of the electrical conductivity of the leachate was carried out because this value provides a quick indication of the amount of ions released from the cement matrix into the pore solution. This in turn influences not only the initial hydration processes and the stability of the microstructure, but also the ability of the composite to conduct electrical current in the saturated state after the hydration processes have been completed. The higher electrical conductivity of the leachate can be an indicator of more suitable conditions for the formation of conductive pathways in the pores, which is key for the design of exterior applications of electro-conductive composites used, for example, in sensing, self-heating or static charge dissipation. For example, granulated blast furnace slag (GGBFS) which is usually used in the construction industry, contains mainly: CaO, SiO_2_, Al_2_O_3_ and MgO in glassy form and releases only limited amounts of conductive ions (Ca^2+^, Mg^2+^, Na^+^, K^+^) into solution, In contrast, the ladle slag produced during secondary steel metallurgy has a higher proportion of free lime, sulphates and iron or manganese oxides, in addition to CaO and SiO_2_, which dissolve easily and release highly electrically conductive ions (Ca^2+^, SO_4_^2-^, Fe^2+^/Fe^3+^, Mn^2+^)^63^. This is also confirmed by the graphical alignment of the values in Fig. 11 below.

According to the values measured above, it is clear that the electrical conductivity of leachates in common Portland and blended cements (CEM I and CEM II) is in the range of ~ 7000–8500 µS/cm, while cements where the primary Portland clinker is replaced by granulated blast furnace slag (CEM III) show lower values (2500 and 5090 µS/cm), which corresponds to their slower hydration and lower ion release and solubility. The impedance of the hydrated composites has a relatively large range of values (720–1110 Ω for CEM I/II and up to 3140 Ω for CEM III) and thus also depends on the pore water that forms the electrolyte that helps to provide the conductive pathways. In contrast, the C/LS-CO_2_ cured system using LS achieves both the highest leachate electrical conductivity (13,940 µS/cm) and the lowest impedance when saturated with water (610 Ω), indicating the formation of stable electrically conductive pathways even in saturated hydrated material. Thus, it can be said that while the blast furnace slag commonly used in blended cements tends to reduce the electrical conductivity, the LS in the C/LS system, on the contrary, provides significantly better electrically conductive properties of the saturated composite. The high ionic conductivity of the C/LS electrolyte makes it the most suitable option for electro-conductive applications outdoors where materials are usually at least partially exposed to moisture. For further experiments it would also be interesting to compare the pore distributions and the internal pore structure.

Interestingly, although CO_2_ curing ‘seals the surface’ in the first stage of carbonation when carbon dioxide reacts with calcium hydroxide dissolved in the pore solution, the composite still has better electrical conductivity in the saturated state than composites without CO_2_ curing. The resulting calcium carbonate crystallizes in the pores as calcite. Due to the size of the molar volume of calcium carbonate and calcium hydroxide, the capillary pores of the cementitious mortar are filled and their permeability decreases, thus also forming a denser material that can potentially conduct electric current instead of the pore solution = the porosity of the cementitious mortar is reduced^46,47,62^.

### Relative ion conductivity of saturated matrix (RIC)

Based on the evaluation of the electrical properties of the solutions and composites, the following table was compiled. The below table has been compiled to quantitatively estimate the conductivity of cement pastes (composite) based on the mobile ion content. Based on available research and publications the assumption used here is that approximately 20% of the total CaO content of the cement contributes to the mobile ions, mainly as Ca^2+^, after hydration^64–66^. This approach is supported by the literature, which indicates that portlandite (Ca(OH)_2_) accounts for approximately 20% of the total CaO in cement after 28 days of hydration^67,68^.

[Ca^2+^] mobile ≈ 0.2 × CaO (wt.%).

The table also includes alkali (Na_2_O and K_2_O) which are electrically conductive, as well as Fe^2^⁺/^3^⁺and an estimated 20% of CaO contributes to mobile Ca^2+^ ions. The differences in the limiting molar conductivities of individual ions (Na⁺, K⁺, Ca^2^⁺, Fe^2^⁺/^3^⁺) are measurable, but relatively small in the context of the cement matrix. The important thing is that these ions provide the main transmission path for electric current in the pore solution. Thanks to them, ionic conductivity is an order of magnitude higher than in the matrix itself, so their mere presence determines the resulting behaviour of the composite in a saturated state, while the differences between individual cations do not play a significant role in practical interpretation.

The presence of Na_2_O, K_2_O and Fe_2_O_3_ was considered for the evaluation of RIC, but these oxides are here as representatives of the corresponding ions (Na⁺, K⁺, Fe^2^⁺/Fe^3^⁺) that can enter the pore solution. Therefore, this is not an exact stoichiometric conversion of the total amount of oxides to free ions, but rather an imaginary part of their content that contributes to ionic conductivity. This method captures the main trend – for comparison in the same type of matrix, see Table 4 below^69^.

- **RIC – relative ion conductivity** = sum of possible partial free conductive ion’s calculation from the equation below
  $${\text{RIC }} = \, ({\text{Na}}_{{2}} {\text{O }} + {\text{ K}}_{{2}} {\text{O }} + { 2}0\% {\text{ of CaO }} + {\text{ Fe}}_{{2}} {\text{O}}_{{3}} )$$

**Table 4:** Comparison of the relative ionic conductivity of composites in the saturated state.

Cement	Na_2_O	K_2_O	Ca^2+^	Fe_2_O_3_	**RIC**
CEM I 42.5R	0.126	0.438	12.85	3.35	**16.61**
CEM II B-M (V-LL) 32.5R	0.419	0.430	9.57	3.38	**13.43**
CEM II BS 32.5R	0.251	0.403	9.74	2.64	**13.10**
CEM II AS 42.5 R	0.422	0.462	10.46	2.86	**13.86**
CEM II A-LL 42.5 R	0.250	0.443	10.93	2.85	**14.30**
CEM II A-S 52.5N	0.147	0.516	10.23	2.30	**13.08**
CEM II A-LL 52.5R	0.351	0.307	10.80	3.15	**14.25**
CEM III B-(LH/RS) 32.5L	0.414	0.471	8.20	1.54	**10.47**
CEM III A 42.5N	0.000	0.453	9.42	1.78	**11.91**
CEM I 42.5R + LS (C/LS)	0.189	0.356	9.66	7.97	**18.05**

According to the relative conductivity in the above table, it follows exactly the trend of electrical conductivity of saturated composites. The highest relative ion conductivity (RIC = 18.05) was determined for the CEM I 42.5R + LS, which also has the lowest impedance (Z) as the saturated composite and also the highest leachate electrical conductivity (ELC) (610 Ω / 13,940 µS/cm). The CEM i 42.5 R (RIC = 16.61) blend has the second highest relative conductivity as calculated with parameters (Z = 690 Ω / ECL = 7770 µS/cm). In contrast, the mixtures CEM III B-(LH/RS) 32.5L (RIC = 10.47 / Z = 3140 Ω / ECL = 2500 µS/cm) and CEM III A 42.5N (RIC = 11.91 / Z = 2160 Ω / ECL = 5090 µS/cm). Based on these results, the relative conductivity can be used to predict the conductivity efficiency of the saturated silicate matrix quite effectively.

### CO_2_ capture balance

The amount of trapped CO_2_ for each CO_2_-cured composite specimen was determined gravimetrically. The samples were weighed after mixing and four hours of hardening and then placed in a CO_2_ chamber where carbonation took place. After the specified time (72 h), the samples were removed from the chamber and reweighed. The difference in weight before and after exposure to CO_2_ corresponds approximately to the amount of carbon dioxide absorbed by the material. The result is approximate mainly due to the possible slight departure of water from the test bodies. Table 5 below shows the weight differences of the composites before and after 72 h CO_2_ curing.

**Table 5:** Comparison of the relative ionic conductivity of composites in the saturated state.

Mixture type	Number sample	m *Fresh* [g]	m *CO*_*2-*_*cured* [g]	Total CO_2_ captured [wt.%]
C/LS CO_2_ W0.40	1	442.05	485.23	**9.77**
	2	439.36	486.13	**10.65**
	3	441.98	488.80	**10.59**
C/LS CO_2_ W0.45	1	431.11	471.80	**9.44**
	2	431.03	479.37	**11.21**
	3	430.10	472.97	**9.97**
C/LS CO_2_ W0.50	1	428.45	467.10	**9.02**
	2	427.68	473.37	**10.68**
	3	429.97	475.22	**10.52**

The results show that these silicate composites are capable of capturing approximately 10 g of CO_2_ on 100 g of composite, which reflects a significant difference from the theoretical calculations reported in other research^42–44^. This ability is mainly related to the diffusion (water vapour resistance factor) of the material and the microstructure—capillaries, pore distribution, etc.

## Conclusion

The present study focused on the comparison of different types of cements and their low carbon alternatives in relation to the electrical properties of cement composites. Particular attention was paid to the impedance of the cured materials as well as the leachate impedance, which reflects the ionic conductivity of the pore solution and its effect on the formation of conductive pathways in the composite.

The results showed that the conventional cements CEM I 42.5R and CEM II A-LL 52.5R achieved the lowest impedance values among the standard binders, while the CEM II blended cements showed slightly better properties in terms of electrical conductivity. In contrast, the use of CEM III B with a high proportion of granulated blast furnace slag resulted in a significant increase in impedance, both in the dry and saturated state, indicating a reduced suitability of this binder for the preparation of electrically conductive composites. This finding corresponds to the lower ionic conductivity of the leachates from these composites, due to the limited ion release typical of GGBFS.

The most promising results were obtained with an alternative cement and slag-based binder system (C/LS) combined with CO_2_ curing. This mixture achieved both the highest leachate conductivity (13,940 µS/cm) and the lowest impedance in the saturated state (610 Ω), indicating the formation of stable and well-developed conductive pathways. The excellent performance of this system can be attributed to the release of highly conductive ions such as Ca^2+^, Fe^2+^/Fe^3+^ and SO_4_^2-^ from the ladle slag as well as the effect of carbonation, which thickens the microstructure but also promotes ion transport under saturated conditions. Thus, from a practical point of view, the C/LS + CO_2_ curing system is particularly suitable for outdoor applications of conductive composites where materials are typically exposed to moisture, such as de-icing, self-heating, sensing or static charge removal.

At the same time, it is important to note that while CO_2_ curing reduces porosity and electrical conductivity in the dry state due to the formation of non-conductive CaCO_3_, it does not adversely affect electrical conductivity in the saturated state. On the contrary, it allows the formation of a more durable and denser matrix, which can enhance both long-term mechanical stability and electrical properties under realistic environmental conditions. This dual effect is of great importance for sustainable construction as it combines CO_2_ sequestration with improved functional properties.

Furthermore, the concept of relative conductivity (RIC) was also introduced, which corresponds to the electrical conductivity parameters of the silicate matrix based on the chemical composition of the binders. This parameter consists of the sum (Na_2_O + K_2_O + 20% of CaO + Fe_2_O_3_) from chemical analysis, relative to the maximum sum of free ions of the compared binders.

The electroconductive silicate material was also found to be able to capture CO_2_ during direct 72 h CO_2_ curing (c = 20%, RH = 60% and t = 28.5 °C) up to approximately 10% by weight of the ECC.

For future research, it would be valuable to investigate in more detail the pore structure and ion transport mechanisms in C/LS composites, especially at different saturation levels and long-term exposure to aggressive environments. Advanced characterization of pore size distribution, connectivity and microstructure evolution during carbonation could provide further insight into the mechanisms controlling electrical conductivity. In addition, optimization of the ratio between Portland cement and ladle slag as well as CO_2_ curing parameters may further improve the balance between mechanical, durability and electrical properties. Another avenue for future research could focus on the use of other alternative raw materials, in particular copper slag, ferrochrome slag, slag from imperial smelters, class F fly ash, which exhibit better strength and durability properties, including the potential endowment of the material with free ions that promote electrical conductivity.

### High lights

- CEM I (ordinary Portland cements) and CEM II (Portland cements with partial replacement by limestone or fly ash)
  o Show relatively low impedance and good ionic conductivity of leachates.
  o Provide favourable electrical conductivity of the composites, especially in saturated state.
  o Suitable as binders for electrically conductive applications.
- CEM III (blast furnace slag cements with high slag content, typically 66–80%)
  o Show much higher impedance and lower leachate conductivity.
  o Due to limited ion release from slag, the formation of conductive pathways is restricted.
  o Less suitable for electro-conductive composites, especially where moisture exposure is expected.
- Alternative binders based on cement + ladle slag (C/LS)
  o Provide the highest ionic conductivity of leachates and lowest impedance in saturated state.
  o Release of conductive ions (Ca^2+^, Fe^2+^/Fe^3+^, Na^+^, K^+^, SO_4_^2-^ ,Mn^2+^) enhances electrical conductivity.
  o CO_2_ curing densifies the microstructure (lower porosity, denser structure) while still maintaining high conductivity when saturated.
  o Promising for outdoor electro-conductive applications (self-heating, sensing, de-icing static charge dissipation).
- Effect of CO_2_-curing on electro conductivity of composites
  o The formation of CaCO₃ during carbonation leads to a solid, non-conducting phase with very low conductivity (also reduces porosity)—corresponding to the high impedance of dry samples after curing.
  o Despite this, the electrical conductivity in the saturated state is slightly better due to more stable ionic pathways in the solid phase (electrically conductive matrix required).
  o Offering an additional environmental benefit by capturing CO_2_ in the material,
  o The tested ECCs are able to capture approximately 10% of the material weight at (72 h, c = 20%, RH = 60% and t = 28.5°C).
- RIC—Relative ion conductivity of saturated matrix
  o This parameter consists of the sum oxides **(Na**_**2**_**O + K**_**2**_**O + 20% of CaO + Fe**_**2**_**O**_**3**_**)** from chemical analysis. Sum of possible free conductive ions of the compared silicate matrixes and reflects the electro-conductivity characteristics of the matrix in a saturated state reasonably reliably.

## Data Availability

The datasets used and/or analysed during the current study available from the corresponding author on reasonable request.
